# Self-Healing Thermoset Polyurethanes Driven by Host–Guest Interactions Between α-Cyclodextrin and Poly(ethylene glycol) Monomethyl Ether or Dodecanol Moieties

**DOI:** 10.3390/molecules30091941

**Published:** 2025-04-27

**Authors:** Riku Miyagawa, Mitsuhiro Shibata

**Affiliations:** Department of Applied Chemistry, Faculty of Engineering, Chiba Institute of Technology, 2-17-1, Tsudanuma, Narashino 275-0016, Chiba, Japan

**Keywords:** self-healing thermoset polyurethane, host–guest interaction, *α*-cyclodextrin, poly(ethylene glycol) monomethyl ether, 1-dodecanol, healing properties, thermal and mechanical properties

## Abstract

Self-healing thermoset polymers have attracted significant attention because they contribute to resource and energy savings by extending their service life. The reactions between glycerol ethoxylate (GCE), *α*-cyclodextrin (*α*-CD), poly(ethylene glycol) monomethyl ether (MPEG), and hexamethylene diisocyanate (HDI) at molar ratios of GCE:*α*-CD:MPEG = *a*:*b*:*c* produced polyurethane networks (GCM-*abc*, *abc* = 311, 411, and 511) containing α-CD and MPEG as host and guest moieties, respectively. To compare this with GCM-411, 1-dodecanol (DN) was used instead of MPEG as a guest molecule to yield a polyurethane network (GCD-411). Dynamic mechanical analysis revealed the formation of a polymer network, and the loss tangent (tan *δ*) peak temperature (*T*_α_) and crosslinking density (*ν*_e_) decreased with increasing GCE fraction for GCMs, and the *T*_α_ and *ν*_e_ values of GCD were slightly higher than those of GCM-411. The tensile strength of the GCMs decreased with increasing GCE fraction, and the tensile strength of GCD-411 was slightly higher than that of GCM-411. All cured films were healed at room temperature for 24 h, and the healing efficiency (*η*_σ_), based on tensile strength, increased in the order of GCM-311 < GCM-411 < GCM-511 < GCD-411. When the healing temperature increased from room temperature to 80 °C, *η*_σ_ increased from 24–38% to 45–62%. GCM-411 and GCD-411 were self-healed thrice by treatment at 80 °C, and *η*_σ_ gradually decreased with each healing cycle.

## 1. Introduction

Thermoset polyurethanes are widely used in flexible and hard foams, coatings, and adhesives because of their excellent chemical and thermal resistances, adhesiveness, and variability in mechanical properties caused by changing the types of isocyanates and polyols. However, cracks and scratches caused by impact or abrasion on the materials using cross-linked polyurethanes cannot be repaired, leading to degradation in their service life. Furthermore, reprocessing and recycling these materials are extremely challenging because they are infusible and insoluble. Therefore, self-healing and reprocessable thermoset polymers have attracted significant attention as resource- and energy-saving materials. Previously, dynamic covalent bonds such as bonds formed by Diels–Alder reaction [1,2,3,4,5], disulfide [6,7,8,9,10,11,12], imine [13,14], phenol-carbamate [15,16,17,18,19,20], boronic ester [20,21,22,23,24], and non-covalent interactions, such as hydrogen bonding [7,11,12,25,26,27,28,29], metal–ligand coordination [10,25,26,27,30], and *π*-*π* interaction [28,29,31], have been utilized to impart self-healing properties to thermoset polyurethanes. Among these, a few studies have demonstrated that damaged samples can self-heal at room temperature (approximately 20–30 °C). However, many samples required high-temperature (typically at 80 to 130 °C) processing. Healing via milder heat-temperature processing (ideally, at room temperature ≈ 20–30 °C; otherwise, at temperatures no higher than 80 °C) is desirable. Self-healing thermoset polyurethanes and epoxy resins, which can be healed via mild-temperature treatments, have been successfully developed using host–guest interactions between *β*-cyclodextrin (*β*-CD) with seven d-glucose units and adamantane (or cyclohexane) moieties [32,33,34]. However, self-healing thermosetting resins driven by host–guest interactions involving *α*-CD (6 d-glucose subunits) have received little attention, even though α-CD forms a pseudo-rotaxane with poly(ethylene glycol) (PEG) [35] and host–guest complexes with 1-dodecy and 1-hexyl compounds [36]. Nakahata et al. reported self-healing hydrogels composed of *α*-CD/PEG-based polyrotaxane and poly(acrylamide-*co*-4-vinylphenylboronic acid) cross-linked with dynamic boronic ester linkages [37]. Cosgun et al. reported self-healing hydrogels composed of α-CD/PEG pseudo polyrotaxane-type complexes, poly(*N*-vinylpyrrolidone) (PVP), and crosslinked poly(ethylene glycol) dimethacrylate networks driven by hydrogen bonding interactions between the α-CD/PEG complexes and PVP [38]. However, these studies focused on hydrogels and not on thermoset polymers, and they did not use reversible complexation of the *α*-CD and PEG moieties as a self-healing mechanism. Very recently, we reported self-healing photo-cured methacrylate resins driven by the reversible formation and dissociation of inclusion complexes in a half-rotaxane (where one end of the axle has a stopper, but the other end is still open) of crosslinked *α*-CD moieties and PEG moieties with only one crosslinked terminal end [39]. However, this methodology has not yet been applied to thermosetting resins, which are widely used for various purposes.

**Scheme 1:**
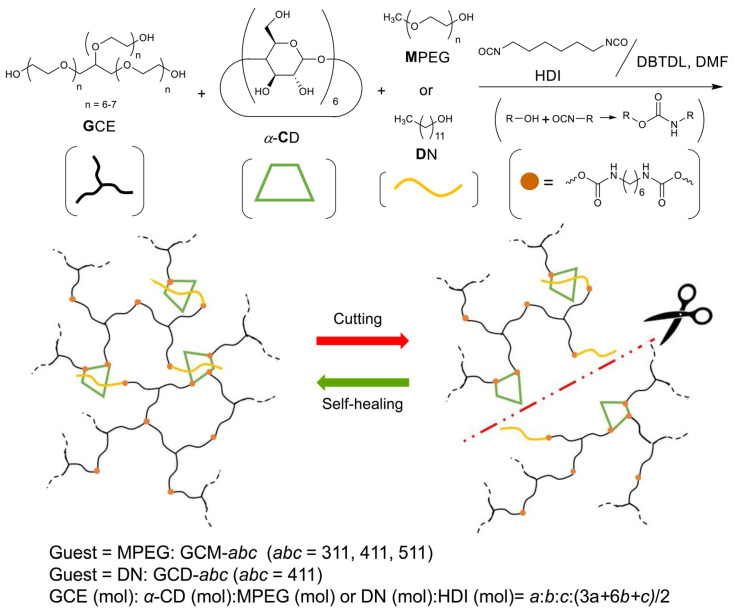
Synthesis of thermoset polyurethanes by the reaction of GCE, *α*-CD, and MOPEG or DN with HDI.

In this study, the reaction of glycerol ethoxylate (GCE), *α*-CD, poly(ethylene glycol) monomethyl ether (MPEG), and hexamethylene diisocyanate (HDI) produced polyurethane networks (GCMs) containing α-CD and MPEG as host and guest moieties, respectively. For comparison with GCM films, a polyurethane network was prepared using 1-dodecanol (DN) instead of MPEG as a guest molecule (GCD-411). In the formed polyurethane networks, reversible formation and dissociation of inclusion complexes in half-rotaxanes of crosslinked *α*-CD moieties and MPEG or DN moieties with only one crosslinked terminal end are expected to impart self-healing properties (Scheme 1). This study focused on the effects of changing the composition of the feed–reactant ratio and using a half-rotaxane of crosslinked *α*-CD/MPEG moieties and a simple inclusion complex of crosslinked *α*-CD/DN moieties on the thermal and mechanical properties, as well as the self-healing ability, of the GCM and GCD films through mild temperature (near-room temperature) processing.

## 2. Results and Discussion

### 2.1. Preparation and Characterization of the GCM and GCD Films

**Figure 1:**
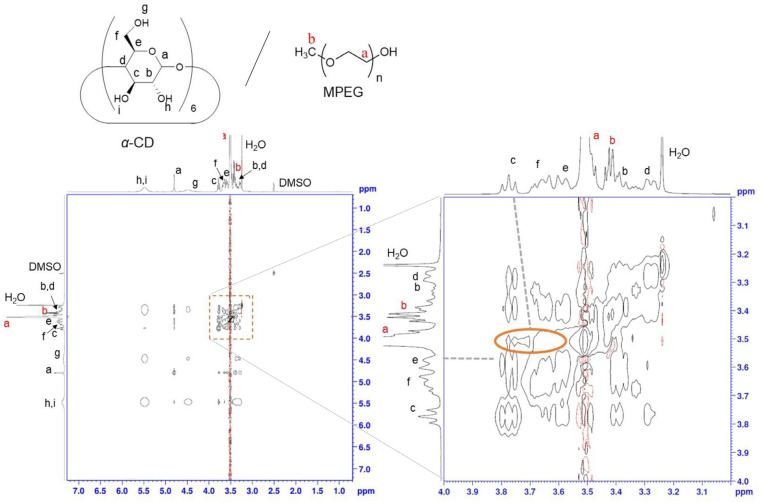
Two-dimensional ^1^H–^1^H NOESY images of the *α*-CD/MPEG mixture with a molar ratio of 1/1 in DMSO-*d*_6_. The concentration of *α*-CD and MPEG is 20 mM.

**Figure 2:**
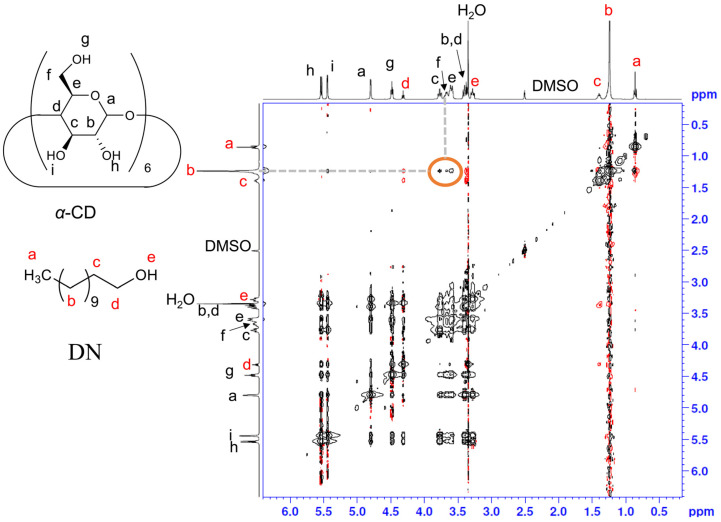
Two-dimensional ^1^H–^1^H NOESY images of the *α*-CD/DN mixture with a molar ratio of 1/1 in DMSO-*d*_6_. The concentration of *α*-CD and DN is 30 mM.

Before the preparation of polyurethane networks from GCE, *α*-CD, MPEG (or DN), and HDI, the formation of a pseudo-rotaxane of *α*-CD/MPEG and an inclusion complex of *α*-CD/DN in DMSO-*d*_6_ was confirmed by 2D ^1^H–^1^H NOESY. The 2D ^1^H–^1^H NOESY images of α-CD/MPEG mixture with a molar ratio of 1/1 in DMSO-*d*_6_ demonstrated a correlation between the H-c, f, and e signals of the α-CD moieties and the H-a signals of the PEG segments of MPEG (Figure 1). The 2D ^1^H–^1^H NOESY images of α-CD/DN mixture with a molar ratio of 1/1 in DMSO-*d*_6_ demonstrated a correlation between the H-c, f, and e signals of the α-CD moieties and the H-b signals of the nonamethylene segments of DN (Figure 2). These results indicate the formation of a pseudo-rotaxane between α-CD and MPEG and the inclusion complex of α-CD and DN. Although these NOESY results do not directly prove the inclusion of α-CD with PEG or DN in the actual cured GCM and GCD films, they provide indirect evidence supporting the occurrence of such inclusion.

The reaction between GCE, *α*-CD, MPEG, and HDI at the molar ratios of 3:1:1:8, 4:1:1:9.5, and 5:1:1:11 produced GCM-311, GCM-411, and GCM-511 films containing α-CD and MPEG as host and guest moieties, respectively. For comparison with GCM-411, DN was used instead of MPEG as the guest molecule to yield a GCD-411 film (Scheme 1). In these reactions, the reaction ratio was determined based on the assumptions that six out of the eighteen hydroxy groups of α-CD and all hydroxy groups of GCE, MPEG, and DN react with the isocyanate groups of HDI in the final composition, and the molar ratio of host–guest is 1/1. GCM-311, GCM-411, and GCM-511 were obtained as homogeneous yellowish transparent films. However, GCD-411 was a yellowish opaque film, suggesting that some hydrophobic DN-derived moieties were microphase-separated (Figure S1, Supplementary Material).

**Figure 3:**
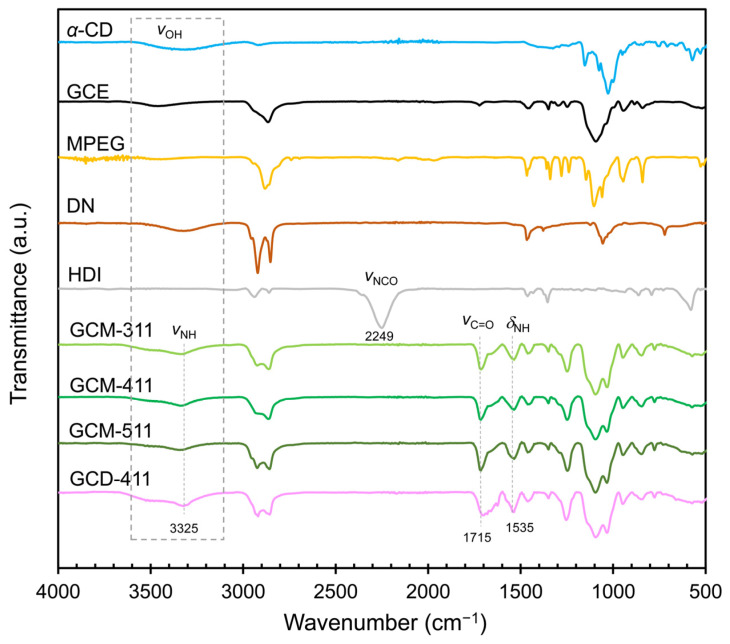
FT-IR spectra of GCE, *α*-CD, MPEG, DN, and all GCM and GCD films.

Figure 3 shows the FT-IR spectra of GCE, *α*-CD, MPEG, DN, HDI, and all GCM and GCD films. In the FT-IR spectra of GCE, *α*-CD, MPEG, and DN, a broad absorption band caused by the OH stretching vibration (*ν*_OH_) was observed at 3100–3600 cm^−1^. HDI exhibited a strong absorption band due to the NCO stretching vibration (*ν*_NCO_) at 2249 cm^−1^. The *ν*_OH_ and *ν*_NCO_ bands, which were observed for GCE, *α*-CD, MPEG, DN, and HDI, were not observed for the GCM and GCD films, and new absorption bands due to NH stretching (*ν*_NH_), urethane C=O stretching (*ν*_C=O_), and NH bending (*δ*_NH_) appeared at 3325, 1715, and 1535 cm^−1^, respectively. These results indicate the occurrence of the urethanization reaction of the hydroxy and isocyanate groups in all cured films. The gel fractions measured using DMF as the extraction and soaking solvents for all cured films were 84–88%, confirming the formation of polyurethane networks.

### 2.2. Thermal Properties of the GCM and GCD Films

**Figure 4:**
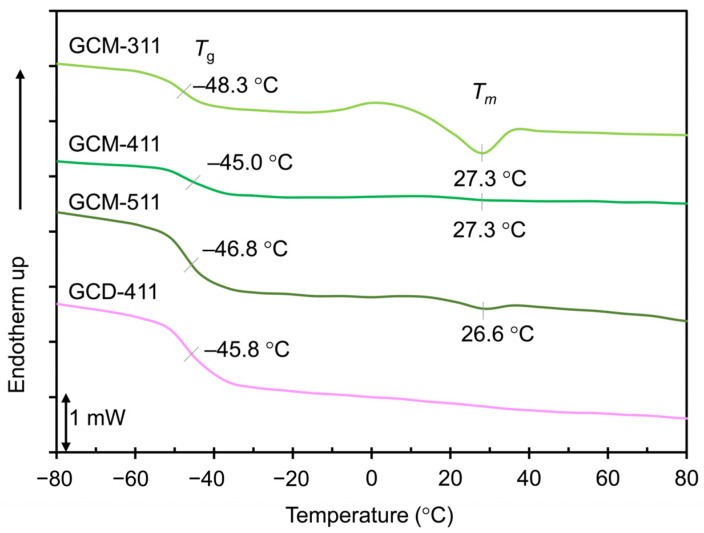
DSC curves of the GCM and GCD films.

Figure 4 shows the DSC curves of the cured films. The *T*_g_s of all cured films ranged from −45 °C to −48 °C, with no significant differences observed. GCM-311, GCM-411, and GCM-511 exhibited an endothermic melting peak at 26–27 °C. As GCD-411 showed no melting peaks, the melting peaks of the GCM films can be attributed to the melting of the PEG segment of MPEG.

**Figure 5:**
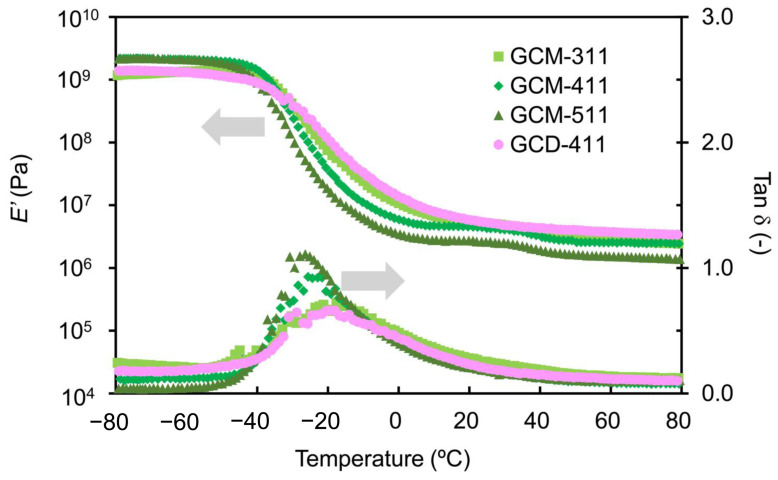
DMA curves of the GCM and GCD films.

Figure 5 shows the DMA curves of the cured films. Table 1 lists *T*_α_, *E*′ at 20 °C, *E*′ at (*T*_α_ + 50) °C, and *ν*_e_ values. The position of the convex tan *δ* curve remained approximately the same for all cured films; this is consistent with the trend of *T*_g_ obtained through DSC measurements. However, *T*_α_ at the peak point of the GCM films slightly decreased with increasing GCE fraction, and *T*_α_ of GCD-411 was higher than that of GCM-411. The *E*′ rubbery plateau region was observed for all cured products at temperatures higher than *T*_α_, indicating the formation of a network structure. The *ν*_e_ value for the GCM films decreased with increasing GCE fraction, and the *ν*_e_ of GCD-411 was higher than that of GCM-411; this is consistent with the trend of *T*_α_. These results can be attributed to the lower hydroxy functionality and longer distances between the terminal hydroxy groups of GCE compared to those of *α*-CD and a much lower feed weight fraction of DN than that of MPEG, causing a lowering of *ν*_e_.

**Figure 6:**
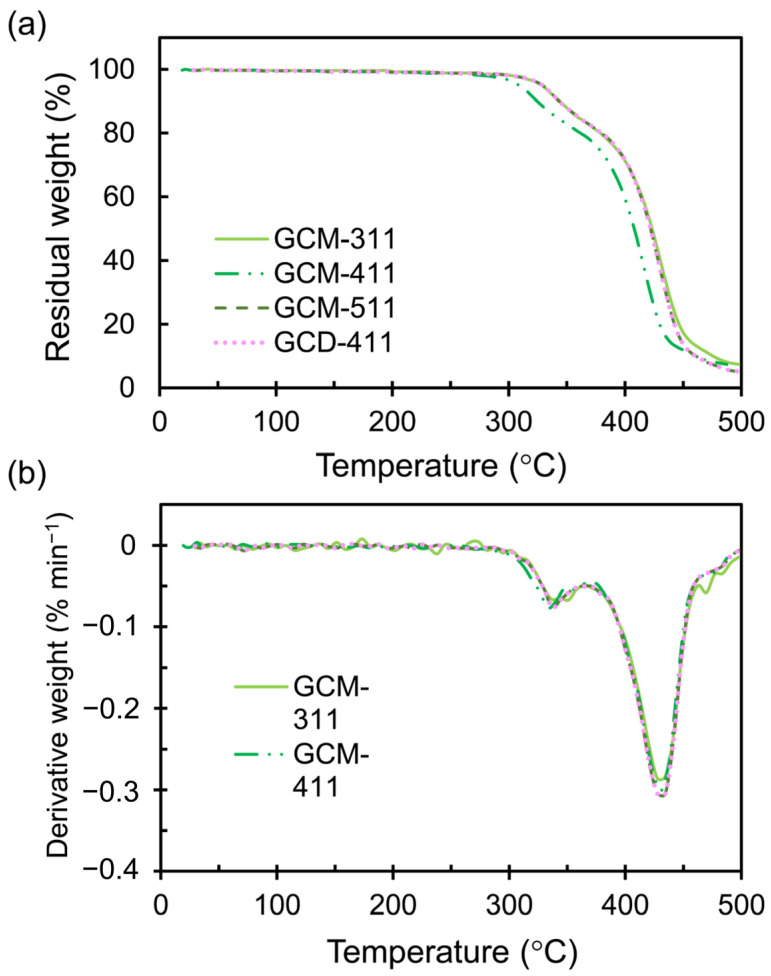
(**a**) TGA and (**b**) DTG curves of the GCM and GCD films.

Figure 6 shows the TGA and DTG curves of the cured films. Table 2 lists the *T*_dp_, *T*_d5%_, *T*_d10%_, and *T*_d50%_ values of the cured films. The DTG curves of the cured films exhibit two distinct degradation steps. The first step at approximately 300–350 °C can be attributed to the thermal decomposition of urethane bonds and *α*-CD-based components, and the second step at approximately 370–460 °C is due to the decomposition of PEG and dodecyl segments. All cured films exhibited *T*_d5%_ values higher than 320 °C, and no significant differences were observed in their *T*_d5%_, *T*_d10%_, and *T*_d50%_ values.

### 2.3. Mechanical and Self-Healing Properties of the GCM and GCD Films

**Figure 7:**
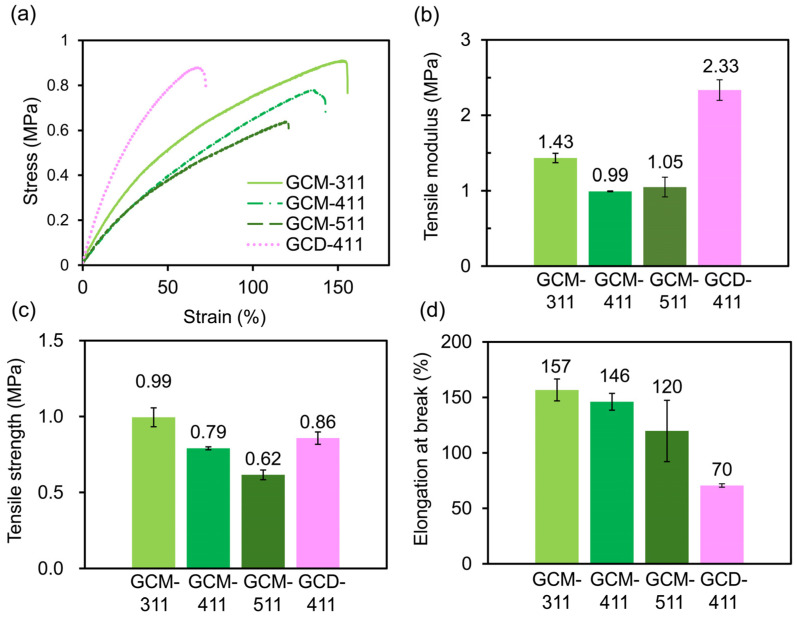
(**a**) Stress–strain curves, (**b**) tensile modulus, (**c**) tensile strength, and (**d**) elongation at break of the cured films.

The mechanical properties of the cured films were evaluated through tensile tests. The stress–strain curves and tensile properties of the cured films are presented in Figure 7. The tensile modulus and tensile strength of the GCM films decreased with increasing GCE content, except for GCM-411 and GCM-511, which exhibited comparable tensile moduli. These results are attributable to the decrease in *ν*_e_ with increasing GCE content. While a reduction in *ν*_e_ typically enhances elongation at break, the GCM films exhibited an opposite trend, showing a decrease in elongation at break with reduced *ν*_e_. The half-rotaxane fraction of α-CD/MPEG contributes to the enhancement of elongation at break due to the sliding motion of the α-CD rings along the MPEG chains. However, in GCE films, the elongation at break decreased with increasing GCE content (that is, decreasing *ν*_e_), which is attributed to the reduction in the half-rotaxane fraction. GCD-411 exhibited a higher tensile modulus and strength and a lower elongation at break than GCM-411, reflecting a higher *ν*_e_ of GCD-411 than that of GCM-411. The α-CD/MPEG half-rotaxane type GCM-411 film exhibited approximately twice the elongation at break compared to the simple inclusion complex type α-CD/DN-based GCD-411.

**Figure 8:**
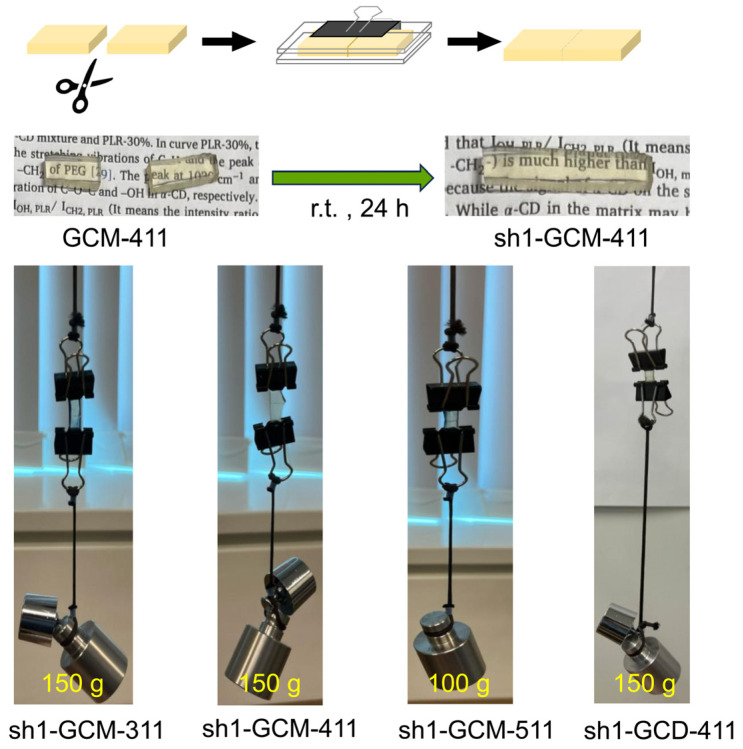
Healing behavior of the GCM and GCD films when kept at room temperature for 24 h.

The as-prepared GCM-411 film was cut into two pieces. The pieces were aligned with their cut surfaces in contact, sandwiched between two polymethylpentene plates, secured with clips, and left at room temperature for 24 h to form healed (sh1) films (Figure 8). All the other cured films were healed using the same method. The self-healed samples of GCM-311, GCM-411, GCM-511, and GCD-411 did not break, even under loadings of 150, 150, 100, and 150 g, respectively.

**Figure 9:**
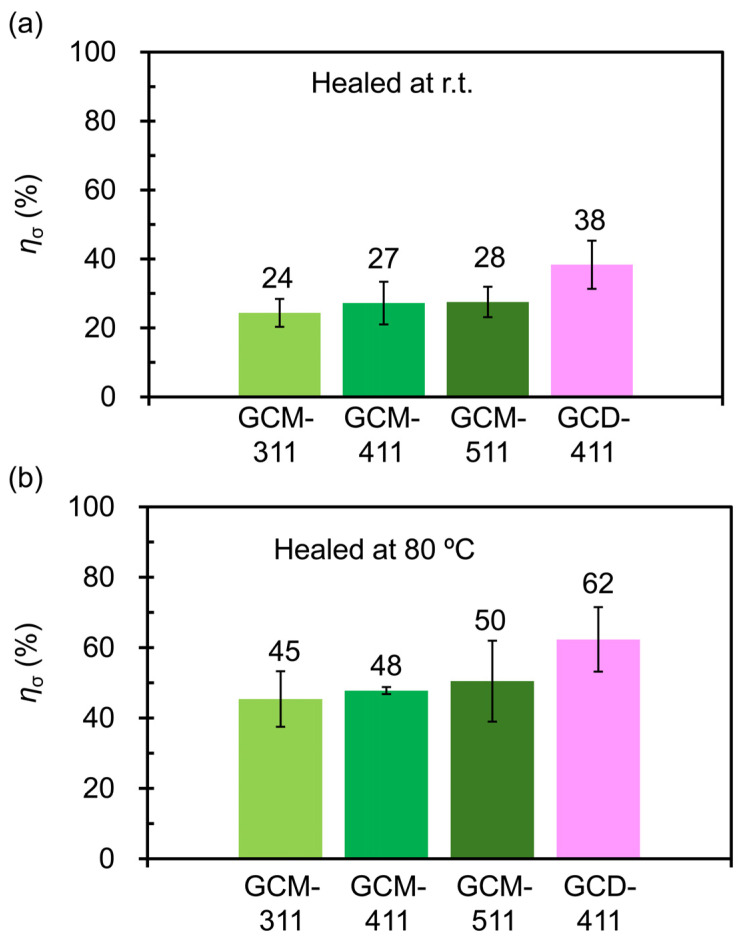
*η*_σ_ of the self-healed GCM and GCD films at (**a**) room temperature and (**b**) 80 °C.

The self-healing properties of the cured products were quantitatively evaluated based on the changes in the tensile strengths of the original and self-healed samples. The stress–strain curves of the original and self-healed samples at room temperature and 80 °C are shown in Figures S2 and S3, respectively. The tensile moduli, tensile strengths, and elongations at break of the original-, sh1-, sh2-, and sh3-GCM and GCD films are listed in Table S1 (Supplementary Material). The *η*_σ_ values at room temperature and 80 °C increased in the order of GCM-311 < GCM-411 < GCM-511 < GCD-411, and the *η*_σ_ values at 80 °C were much higher than those at room temperature (Figure 9). The *η*_σ_ values of the GCM films increased with increasing GCE fraction, even though the α-CD/MPEG half-rotaxane fraction decreased. This result can be attributed to the decrease in the *E*’ values in the temperature range of 20 °C to 80 °C, promoting reversible host–guest interactions between the α-CD and MPEG moieties. At room temperature, the *η*_σ_ value of GCM-511 (28%) was higher than that of our previously reported analog polyurethane network (17%), which was synthesized by the reaction of GCE, β-CD, 1-adamantanol, and HDI at a molar ratio of 5:1:1 (GCE:β-CD:1-adamantanol) [32]. The fact that the *η*_σ_ of GCD-411 was higher than that of GCM-411 suggests that the reversible host–guest interactions between α-CD and relatively hydrophobic dodecyl components are stronger than those between α-CD and relatively hydrophilic MPEG components.

**Figure 10:**
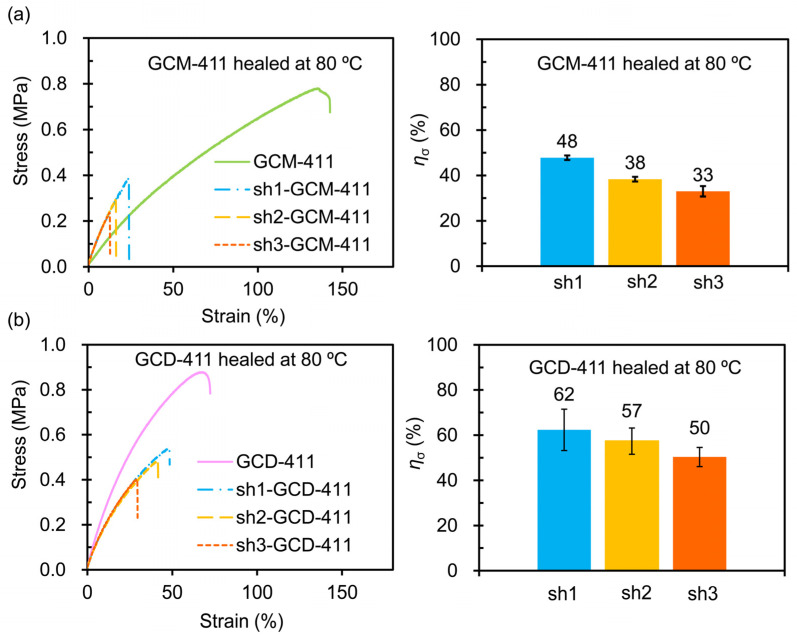
Changes in the stress–strain curves and *η*_σ_ values caused by repetitive healing (sh1, sh2, and sh3) at 80 °C for (**a**) GCM-411 and (**b**) GCD-411.

We investigated the repetitive self-healing properties by treating the GCM-411 and GCD-411 films at 80 °C. Figure 10 shows the stress–strain curves and *η*_σ_ values of the GCM-411 and GCD-411 films, which were healed thrice at 80 °C for 24 h. Comparing the stress–strain curves of the original and healed GCM-411 samples, the maximal stress and strain at break decreased with an increasing number of healing cycles, and the initial slope (i.e., tensile modulus) of the self-healed samples was higher than that of the original GCM-411. However, the tensile modulus of the GCM-411 film healed at room temperature was almost the same as that of the original GCM-411 film as shown in Figure S2 and Table S1. Although the exact mechanism remains unclear, annealing the damaged GCM-411 film at 80 °C followed by natural cooling to room temperature led to the recrystallization of the melted crystalline MPEG segments. This process may also have altered the crystalline state of MPEG segments and hydrogen bonding interactions of the polyurethane network. Conversely, comparing the stress–strain curves of the original and healed amorphous GCD-411 samples, the maximal stress and strain at break decreased with an increasing number of healing cycles, but the initial slope (i.e., tensile modulus) of the self-healed samples was lower than that of the original GCM-411. The *η*_σ_ values of the GCM-411 and GCD-411 gradually decreased owing to the thrice healing from 48% to 33% and 62% to 50%, respectively.

## 3. Materials and Methods

### 3.1. Materials

This study procured *α*-CD (purity > 98.0%) and dibutyltin dilaurate (DBTDL, purity > 95.0%) from Kanto Chemical Co. Inc. (Tokyo, Japan), GCE (*M*_n_: ~1000, degree of polymerization: 20.4) from Sigma-Aldrich Japan Co., Ltd. (Tokyo, Japan), and MPEG (average molecular weight: 950–1050), DN (purity > 99.0%), and HDI (purity > 99.0%) from Tokyo Chemical Industry (Tokyo, Japan). All commercially available reagents were used as received without further purification.

### 3.2. Preparation of Polyurethane Network Films

A typical procedure for GCM-411 [molar ratio of GCE: *α*-CD:MPEG:HDI = 4:1:1:(3∙4 + 6∙1 + 1)/2] is as follows. HDI (0.356 g, 2.12 mmol) is added to a solution of GCE (4.20 g, 4.24 mmol) and DBTDL (40 mg, 0.5 wt.% of total weight of reactants) in dehydrate *N*,*N*-dimethyl formamide (DMF, 5 mL) and stirred at room temperature for 30 min in a nitrogen atmosphere. A solution of MPEG (1.06 g, 1.06 mmol) in dehydrated DMF (5 mL) is added to the reaction mixture and stirred at room temperature for 30 min in a nitrogen atmosphere; subsequently, HDI (0.0891 g, 0.530 mmol) is added and stirred at room temperature for 30 min in a nitrogen atmosphere. Then, *α*-CD (1.03 g, 1.06 mmol) in dehydrated DMF (10 mL) is added to the reaction mixture and stirred at room temperature for 30 min in a nitrogen atmosphere; subsequently, HDI (1.25 g, 7.41 mmol) is added and stirred at room temperature for 30 min in a nitrogen atmosphere. The resulting solution is poured into a culture dish composed of perfluoroalkoxy alkanes, sonicated at room temperature for 10 min, and dried at 40 °C for 24 h in an electric oven and at 100 °C for 24 h in a vacuum oven to produce the GCM-411 film. During this reaction, it is assumed that the highly reactive six primary hydroxy groups among the 18 hydroxy groups of *α*-CD react with the isocyanate groups of HDI [40,41]. GCM-311, GCM-511, and GCD-411 were prepared following a procedure similar to that used to prepare GCM-411. The amounts of feed reactants for all cured products are listed in Table 3.

### 3.3. Self-Healing Experiments and Analyses

Rectangular samples (20 × (4–5) × (1.6–2.6) mm^3^) of the GCM and GCD films were cut into halves, and the two halves were aligned with their cut surfaces in contact, sandwiched between two polymethylpentene plates, secured with clips, and left at room temperature or 80 °C for 24 h to produce self-healing GCM and GCD films (sh1-GCM and sh1-GCD, respectively). This healing cycle was repeated thrice (sh1, sh2, and sh3). Healing efficiency was evaluated by calculating the tensile strength recovery rate using the following equation:

*η*_σ_ (%) = 100*σ*_1_/*σ*_0_ (1)

where *σ*_0_ and *σ*_1_ are the average tensile strengths of the original and healed samples, respectively.

### 3.4. Measurements

Two-dimensional (2D) ^1^H–^1^H nuclear Overhauser effect spectroscopy (2D ^1^H–^1^H NOESY) was performed, and the resulting spectra were recorded at 300 K using a Bruker AV-400 (400 MHz) instrument (Billerica, MA, USA), with DMSO-*d*_6_ as the solvent and tetramethylsilane (TMS) as the internal standard. Fourier-transform infrared (FT-IR) spectra were recorded using a Shimadzu (Kyoto, Japan) IRAffinity-1S instrument in the 500–4000 m^−1^ range using the attenuated total reflectance method. The FT-IR spectra were acquired using 50 scans at a resolution of 4 cm^−1^. Gel fraction measurements were performed using the following procedure. After a film sample (10 × 10 × (1.6–2.6) mm^3^) was soaked in DMF at room temperature (20–25 °C) for 24 h, the film sample was vacuum-dried at 100 °C for 24 h. The gel fraction was calculated as follows:

*Gel fraction* (%) = 100 *w*_1_/*w*_0_ (2)

where *w*_0_ and *w*_1_ are the weights of the original and dried films, respectively. Three samples of each film were tested, and the mean values and standard deviations were calculated from the gel fraction measurement. Differential scanning calorimetry (DSC) was performed using a Shimadzu DSC-60 Plus instrument (Shimadzu Corp., Kyoto, Japan) in a nitrogen atmosphere. After the as-prepared sample (8–9 mg) was cooled to −80 °C, the heating scan was monitored at a heating rate of 20 °C min^−1^. The glass transition temperature (*T*_g_) was determined from the midpoint of the heat flow change. Dynamic mechanical analysis (DMA) (DMA1, Mettler–Toledo, Tokyo, Japan) was performed on a rectangular plate sample (20 × (4–5) × (1.6–2.2) mm^3^) with a chuck distance of 10 mm, a frequency of 1 Hz, and heating rate of 3 °C min^−1^. The amplitude of the DMA measurements was 7 μm. *T*_α_ was obtained from the temperature dependency of tan *δ*. The crosslinking densities (*ν*_e_) of the cured products were calculated using the following equation:

*ν*_e_ = *E′*/(3*RT*), (3)

where *R* is the gas constant (8.314 J mol K^−1^), *E′* is the storage modulus of the cured film in the rubbery region at (*T*_α_ + 50) K, and *T* is the absolute temperature at which the storage modulus values are obtained [42,43]. Thermogravimetric analysis (TGA) and derivative thermogravimetric analysis (DTG) were performed for a sample weighing approximately 3–5 mg using a Shimadzu DGA-60 thermogravimetric analyzer at a heating rate of 20 °C min^−1^ in a nitrogen atmosphere. The temperatures at which *x*% mass loss occurred (*T*_d*x*%_, *x* = 5, 10, and 50) and the decomposition peak temperature (*T*_dp_) were determined using the TGA and DTG curves, respectively. The tensile tests of the rectangular samples (20 × (4–5) × (1.6–2.6) mm^3^) were performed at a temperature of 20–25 °C using a Shimadzu Autograph AG-I instrument. The span length and testing speed were 15 mm and 3 mm min^−1^, respectively. Five specimens were tested for each set of samples, and the mean values and standard deviations were calculated.

## 4. Conclusions

The reactions between GCE, CD, MPEG, and HDI at the molar ratios of 3:1:1:8, 4:1:1:9.5, and 5:1:1:11 produced GCM-311, 411, and 511, respectively, containing reversible α-CD/MPEG half-rotaxane structures. Compared to GCM-411, when DN was used as a guest molecule instead of MPEG, GCD-411 containing a reversible αCD/DN host–guest inclusion complex structure was obtained. The ^1^H–^1^H NOESY images of α-CD/MPEG and α-CD/DN mixtures in DMSO-*d*_6_ demonstrated the formation of a pseudo-rotaxane and an inclusion complex between α-CD and MPEG and α-CD and DN, respectively. FT-IR and gel fraction analyses of all cured films revealed the formation of polyurethane networks. DSC analysis revealed a melting endotherm owing to the crystallized MPEG segments for the GCM films, whereas GCD-411 was amorphous. DMA analyses revealed that *T*_α_ and *ν*_e_ decreased with increasing GCE fraction for the GCM films, and the *T*_α_ and *ν*_e_ values of GCD-411 were slightly higher than those of GCM-411. TGA and DTG analyses revealed that the *T*_d5%_ values of all cured films were higher than 320 °C, and all cured films showed two-step thermal degradation. The tensile strength of the GCM films decreased with increasing GCE content, and the tensile strength of GCD-411 was slightly higher than that of GCM-411. All cured films were healed at room temperature for 24 h, and the *η*_σ_ values increased in the order of GCM-311 < GCM-411 < GCM-511 < GCD-411. By increasing the healing temperature from room temperature to 80 °C, *η*_σ_ increased from 24–38% to 45–62%. GCM-411 and GCD-411 were self-healed thrice by treatment at 80 °C, and *η*_σ_ gradually decreased as the healing cycle was repeated. Because the GCM and GCD films developed in this study exhibited excellent self-healing properties and good thermal and mechanical properties, they are expected to be applied to environmentally benign coatings.

## Figures and Tables

**Table 1:** *T*_α_, *E’* at 20 °C, *E’* at (*T*_α_ + 50) °C, and *ν*_e_ values measured via DMA for the cured films.

Sample	*T*_α_ (°C)	*E’* (MPa) at 20 °C	*E’* (MPa) at (*T*_α_ + 50) °C	*ν*_e_ (mmol cm^−3^)
GCM-311	−17.6	55.3	4.59	0.602
GCM-411	−25.6	4.54	4.29	0.579
GCM-511	−26.4	2.73	2.61	0.353
GCD-411	−19.3	5.77	4.75	0.627

**Table 2:** *T*_d5%_, *T*_d10%_, and *T*_d50%_ values of the GCM and GCD films.

Sample	*T*_dp_ (°C)	*T*_d5%_ (°C)	*T*_d10%_ (°C)	*T*_d50%_ (°C)
GCM-311	341, 433	331	346	423
GCM-411	334, 430	327	341	422
GCM-511	338, 430	332	346	422
GCD-411	357, 432	321	345	424

**Table 3:** Amounts of feed reactants of the cured products.

Sample	GCE/HDI *^1^		MPEG or DN/HDI		*α*-CD/HDI *^1^	
	GCE *^2^ g (mmol)	HDI g (mmol)	MPEG or DN *^3^ g (mmol)	HDI g (mmol)	*α*-CD *^4^ g (mmol)	HDI g (mmol)
GCM-311	3.77 (3.80)	0.320 (1.90)	1.27 (1.27)	0.110 (0.630	1.23 (1.27)	1.28 (7.61)
GCM-411	4.20 (4.24)	0.356 (2.12)	1.06 (1.06)	0.0891 (0.530)	1.03 (1.06)	1.25 (7.41)
GCM-511	4.50 (4.54)	0.382 (2.27)	0.908 (0.908)	0.0764 (0.454)	0.883 (0.908)	1.22 (7.28)
GCD-411	4.72 (4.76)	0.400 (2.38)	0.119 (1.19)	0.1000 (0.595)	1.16 (1.19)	1.40 (8.33)

*^1^ OH/NCO molar ratios for GCE/HDI, MPEG/HDI (or DN/HDI), and *α*-CD/HDI are 3/1, 1/1, and 3/2, respectively. The amount of HDI for MPEG or DN/HDI is set when OH/NCO molar = 1/1. The reaction ratio is determined based on the assumptions that six primary hydroxy groups of α-CD and all hydroxy groups of GCE, MPEG, and DN react with the isocyanate groups of HDI in the final composition, and the molar ratio of host/guest is 1/1. *^2^ The molecular weight of the GCE is calculated to be 991, based on the degree of polymerization (20.4). *^3^ The molar amounts of MPEG and DN are calculated based on their molecular weights (1000 and 186.3, respectively). MPEG is used for GCM-311, GCM-411, and GCM-511, and DN is used for GCD-411. T. *^4^ The molar amount of *α*-CD is calculated based on its molecular weight (972.9).

## Data Availability

All data are available in the manuscript.

## Supplementary Materials

The following supporting information can be downloaded online: https://www.mdpi.com/article/10.3390/molecules30091941/s1, Figure S1. Appearance of cured films. Figure S2. Stress–strain curves of original and once-self-healed films at room temperature for 24 h. Figure S3. Stress–strain curves of original and once-self-healed films at 80 °C for 24 h. Table S1. Tensile moduli, tensile strengths, and elongation at breaks of original-, sh1-, sh2-, and sh3-GCM and GCD films.

## References

[1] Yang S., Du X., Deng S., Qiu J., Du Z., Cheng X., Wang H. (2020). Recyclable and self-healing polyurethane composites based on Diels-Alder reaction for efficient solar-to-thermal energy storage. Chem. Eng. J..

[2] Du W., Jin Y., Shi L., Shen Y., Lai S., Zhou Y. (2020). NIR-light-induced thermoset shape memory polyurethane composites with self-healing and recyclable functionalities. Compos. B.

[3] Wu P., Cheng H., Wang X., Shi R., Zhang C., Arai M., Zhao F. (2021). A self-healing and recyclable polyurethane-urea Diels–Alder adduct synthesized from carbon dioxide and furfuryl amine. Green Chem..

[4] Yang T., Lin C., Huang M., Ying P., Zhang P., Wu J., Wang T., Kovalev A., Myshkin N., Levchenko V. (2024). Self-healing and recyclable polyurethane/nanocellulose elastomer based on the Diels-Alder reaction. Polymers.

[5] Yang B., Chen X., Li Y., Ruan H. (2024). Thermal-driven self-healing and recyclable thermosetting polyurethane resins for energy harvesting. Eur. Polym. J..

[6] Ye J., Liu H., Zhu D., Guo C., Liu Y., Feng L. (2025). Multiple responsive self-healing behavior of amino-functionalized CuS-modified thermo-reversible polyurethane containing double dynamic covalent bonds. Eur. Polym. J..

[7] Liu M., Zhong J., Li Z., Rong J., Yang K., Zhou J., Shen L., Gao F., Huang X., He H. (2020). A high stiffness and self-healable polyurethane based on disulfide bonds and hydrogen bonding. Eur. Polym. J..

[8] He J., Song F., Li X., Gong X., Tu W. (2021). A novel kind of room temperature self-healing poly(urethane-urea) with robust mechanical strength based on aromatic disulfide. J. Polym. Res..

[9] Ye G., Jiang T. (2021). Preparation and properties of self-healing waterborne polyurethane based on dynamic disulfide bond. Polymers.

[10] Zhang W., Wang M., Zhou J., Sheng Y., Xu M., Jiang X., Ma Y., Lu X. (2021). Preparation of room-temperature self-healing elastomers with high strength based on multiple dynamic bonds. Eur. Polym. J..

[11] Ma J., Pang X., Chen L., Qiu P. (2025). Super adhesive, self-healing elastomer based on synergistic dual dynamic interactions for corrosion-resistant coatings. Appl. Mater. Today.

[12] Fu B., Wu Y., Cao X., Wei K., Shan B. (2025). Eco-friendly fabrication of self-healing robust waterborne polyurethane based on dual dynamic networks for metal corrosion protection. Prog. Org. Coat..

[13] Fan W., Jin Y., Shi L., Zhou R., Du W. (2020). Developing visible-light-induced dynamic aromatic Schiff base bonds for room-temperature self-healable and reprocessable waterborne polyurethanes with high mechanical properties. J. Mater. Chem. A.

[14] Xie D.-M., Lu D.-X., Zhao X.-L., Li Y.D., Zeng J.-B. (2021). Sustainable and malleable polyurethane networks from castor oil and vanillin with tunable mechanical properties. Ind. Crop. Prod..

[15] Naveed M., Rabnawaz M., Khan A., Tuhin M.O. (2019). Dual-layer approach toward self-healing and self-cleaning polyurethane thermosets. Polymers.

[16] Li M., Ding H., Yang X., Xu L., Xia J., Li S. (2020). Preparation and properties of self-healing polyurethane elastomer derived from tung-oil-based polyphenol. ACS Omega.

[17] Ding H., Yang X., Xu L., Li S., Xia J., Li M. (2020). Thermally reversible, self-healing polyurethane based on propyl gallate and polyurethane prepolymers with varied isocyanate content. J. Renew. Mater..

[18] Xu X., Ma X., Cui M., Zhao H., Stott N., Zhu J., Yan N., Chen J. (2024). Fully biomass-derived polyurethane based on dynamic imine with self-healing, rapid degradability, and editable shape memory capabilities. Chem. Eng. J..

[19] Kubota R., Shibata M. (2024). Healable thermoset polyurethanes with high biomass content driven by dynamic phenol-carbamate bonds. Polym. Bull..

[20] Kubota R., Shibata M. (2025). Bio-based healable thermoset polyurethanes containing dynamic phenol–carbamate bonds derived from quercetin and poly(trimethylene glycol). J. Polym. Res..

[21] Yang Y., Du F.-S., Li Z.-C. (2020). Highly stretchable, self-healable, and adhesive polyurethane elastomers based on boronic ester bonds. ACS Appl. Polym. Mater..

[22] Song K., Ye W., Gao X., Fang H., Zhang Y., Zhang G., Li X., Yang S., Wei H., Ding Y. (2021). Synergy between dynamic covalent boronic ester and boron–nitrogen coordination: Strategy for self-healing polyurethane elastomers at room temperature with unprecedented mechanical properties. Mater. Horiz..

[23] Li J., Hu C., Yang B., Ning Z., Zeng Y. (2022). Recyclable, self-healing itaconic acid-based polyurethane networks with dynamic boronic ester bonds for recoverable adhesion application. Polymer.

[24] Liu Y., Zhang J., Ji Y., Cao J., Xu S., Luo P., Liu J., Ma L., Gao G., Wu Y. (2025). Self-healing polyurethane elastomers with dynamic crosslinked networks for complex structure 3D printing. Chem. Eng. J..

[25] Xu J., Wang X., Zhang X., Zhang Y., Yang Z., Li S., Tai L., Wang Q., Wang T. (2023). Room-temperature self-healing supramolecular polyurethanes based on the synergistic strengthening of biomimetic hierarchical hydrogen-bonding interactions and coordination bonds. Chem. Eng. J..

[26] Xu G., Liang Z., Huang Q., Wang Y., Yang J., Nie Y. (2023). Tough self-healing polyurethane elastomers based on interpenetrating networks containing multiple hydrogen bond networks, flexible blocks, metal coordination and covalent cross-linking. Prog. Org. Coat..

[27] Su T.-Y., Su L.-W., Zhao Z., Xing R.-G., Ge X., Pan G.-F., Bao J.-X. (2024). High Stretchability and Elastic Self-Healing Polyurethane Elastomer through Dual Cross-Linking with Metal−Ligand Coordination and hydrogen bonds. ACS Appl. Polym. Mater..

[28] Yuan X., Lin X., Dong F., Huang X., Liu H., Xu X. (2025). Self-healed, tough, and highly resilient elastomer facilitated by cooperative hydrogen-bonding interaction and π−π stacking interaction. ACS Appl. Polym. Mater..

[29] Jiang H., Yan T., Cheng M., Zhao Z., He T., Wang Z., Li C., Sun S., Hu S. (2025). Autonomous self-healing and superior tough polyurethane elastomers enabled by strong and highly dynamic hard domains. Mater. Horiz..

[30] Park H., Kang T., Kim H., Kim J.-C., Bao Z., Kang J. (2023). Toughening self-healing elastomer crosslinked by metal–ligand coordination through mixed counter anion dynamics. Nat. Commun..

[31] Wu P.H., Xie C.H., Li Y.Q., Huang C.J., Xie H.B., You Y. (2025). Fatigue-resistant, self-healable and thermally conductive polyurethane composites based on the intrinsic π-π stacking interactions between boron nitrides and hard segments. Mater. Today Commun..

[32] Sugane K., Shibata M. (2021). Self-healing thermoset polyurethanes utilizing host–guest interaction of cyclodextrin and adamantane. Polymer.

[33] Sekiya T., Shibata M. (2023). Self-healing castor oil-based polyurethane networks featuring cyclodextrin–adamantane host–guest interactions. Polym. Bull..

[34] Kurihara R., Ogawa Y., Sugane K., Shibata M. (2024). Self-healing carboxylic acid-cured epoxy networks driven by the cyclodextrin–cyclohexane host–guest interaction. Polym. Bull..

[35] Harada A., Kamachi M. (1990). Complex formation between poly(ethylene glycol) and α-cyclodextrin. Macromolecules.

[36] Yamaguchi H., Kobayashi R., Takashima Y., Hashidzume A., Harada A. (2011). Self-assembly of gels through molecular recognition of cyclodextrins: Shape selectivity for linear and cyclic guest molecules. Macromolecules.

[37] Nakahata M., Mori S., Takashima Y., Yamaguchi H., Harada A. (2016). Self-healing materials formed by cross-linked polyrotaxanes with reversible bonds. Chem.

[38] Cosgun S.N.K., Tuncaboylu D.C. (2021). Cyclodextrin-linked PVP/PEG supramolecular hydrogels. Carbohydr. Polym..

[39] Honma Y., Sugane K., Shibata M. (2024). Thermal, mechanical, and self-healing properties of polymer networks produced by photo-polymerizing α-cyclodextrin-glycidyl methacrylate adduct and poly(ethylene glycol) methacrylate. J. Polym. Res..

[40] Khan A.R., Forgo P.F., Stine K.J., D’Souza V.T. (1998). Methods for selective modifications of cyclodextrins. Chem. Rev..

[41] Mandal S.S., Choudhury A.M., Gupta A., Maiti P. (2025). An injectable cyclodextrin extended polyurethane/carboxymethyl cellulose hydrogel for controlled release of insulin: In-vitro and in-vivo diabetic animal model study. Carbohyd. Polym..

[42] Hill L.W. (1997). Calculation of crosslink density in short chain networks. Prog. Org. Coat..

[43] Echeverria-Altuna O., Ollo O., Larraza I., Gabilondo N., Harismendy I., Eceiza A. (2022). Effect of the biobased polyols chemical structure on high performance thermoset polyurethane properties. Polymer.

