# Supplementary material for: Self-Healing Thermoset Polyurethanes Driven by Host–Guest Interactions Between α-Cyclodextrin and Poly(ethylene glycol) Monomethyl Ether or Dodecanol Moieties
Source: Molecules. 2025 Apr 27;30(9):1941. doi: 10.3390/molecules30091941 (PMC12073313; doi:10.3390/molecules30091941)
Supplement: Supplementary file 1 [file molecules-30-01941-s001.zip › molecules-3595685-supplementary.pdf]

Supplementary Material

Self-Healing Thermoset Polyurethanes Driven by the Host-Guest Interaction Between α-Cyclodextrin and Poly(ethylene glycol) Monomethyl Ether or Dodecanol Moieties

Riku Miyagawa, Mitsuhiro Shibata

[1] Department of Applied Chemistry, Faculty of Engineering,
Chiba Institute of Technology, 2-17-1, Tsudanuma, Narashino, Chiba 275-0016, Japan

*Corresponding author.
E-mail address: mitsuhiro.shibata@p.chibakoudai.jp (M. Shibata)
ORCID: 0000-0003-1341-3712

Contents

Figure S1. Appearance of the cured films.

Figure S2. Stress-strain curves of the original and self-healed GCM and GCD films at room temperature.

Figure S3. Stress-strain curves of the original and self-healed GCM and GCD films at 80 °C.

Table S1. Tensile moduli, tensile strengths and elongation at breaks of original-, sh1-, sh2-, and sh3-GCM and GCD films.

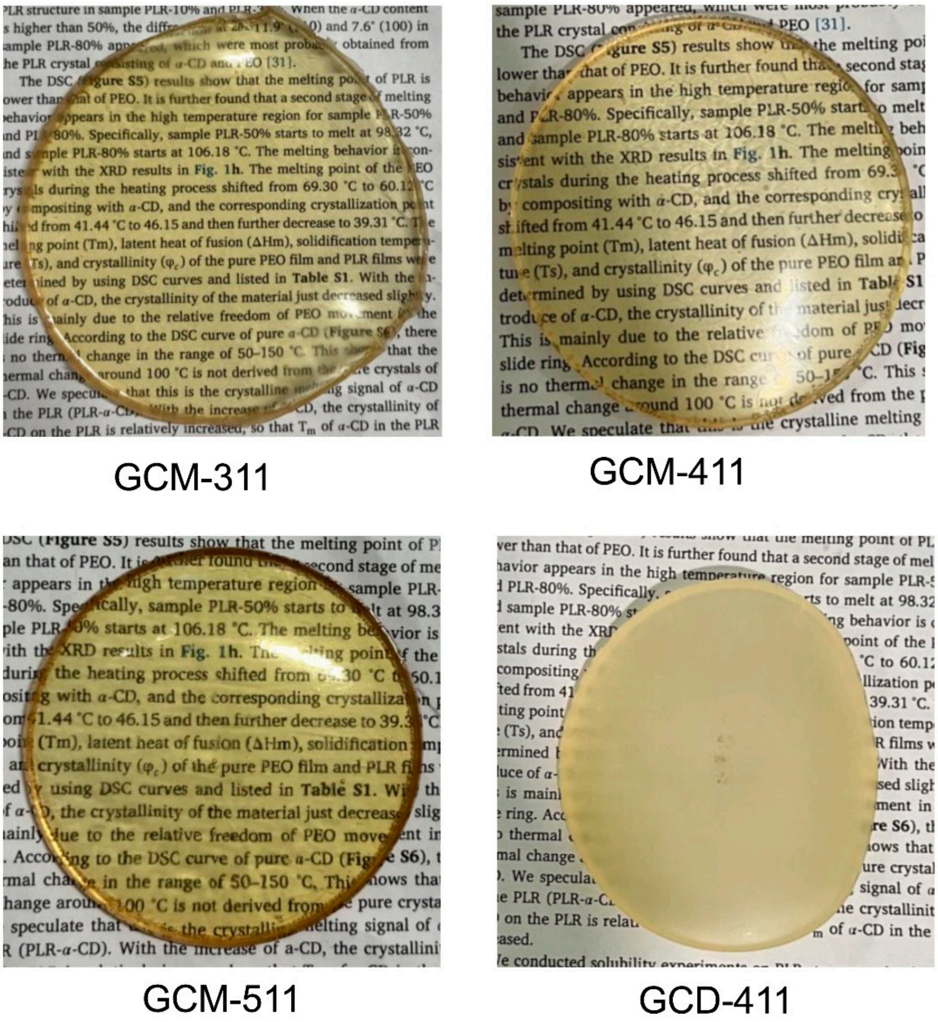

Figure S1. Appearance of the cured films.

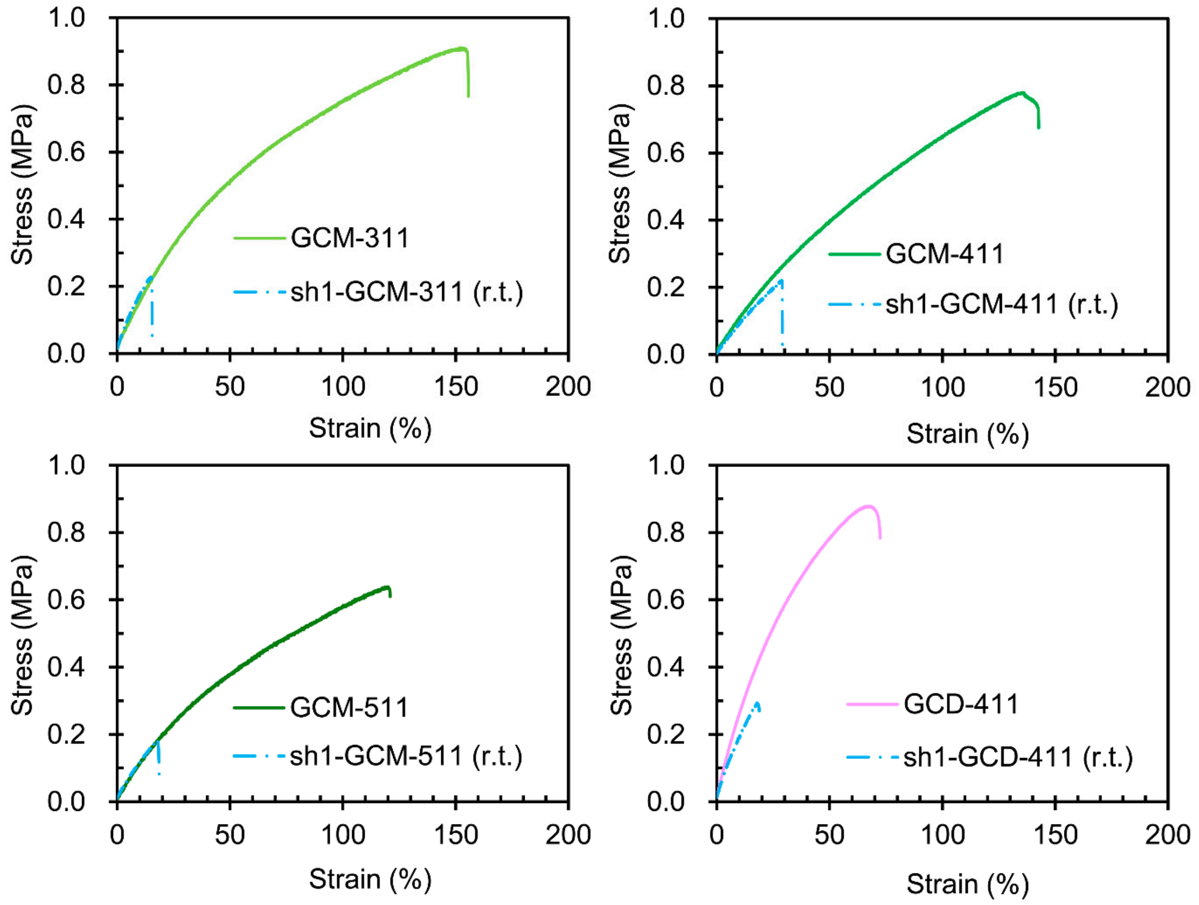

Figure S2. Stress-strain curves of the original and self-healed GCM and GCD films at room temperature.

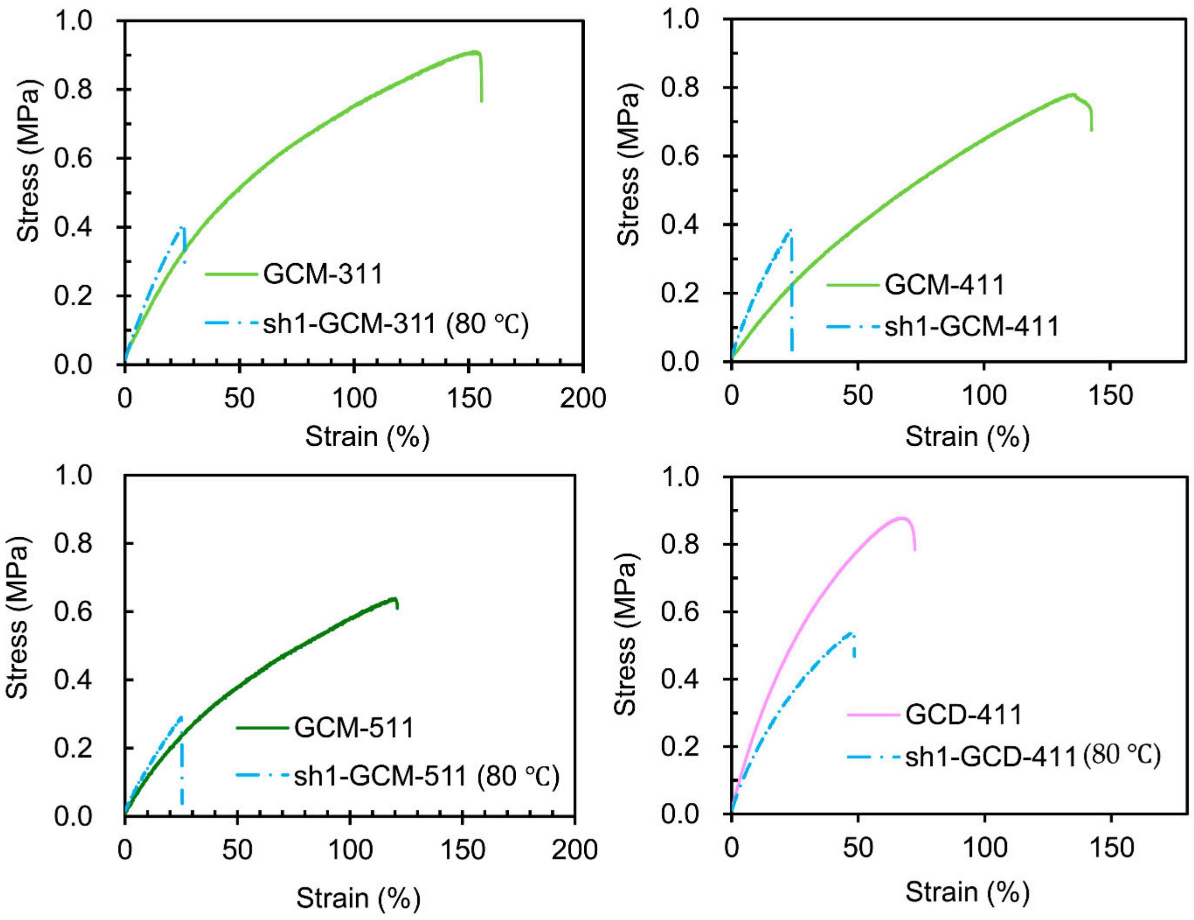

Figure S3. Stress-strain curves of the original and self-healed GCM and GCD films at 80 °C.

Table S1. Tensile moduli, tensile strengths and elongation at breaks of original-, sh1-, sh2-, and sh3-GCM and GCD films.

Sample	Healing temperature (°C)	Tensile modulus (MPa)	Tensile strength (MPa)	Elongation at break (%)
GCM-311	-	1.43 ± 0.15	0.99 ± 0.06	157 ± 10
sh1-GCM-311	r.t.	1.00 ± 0.17	0.12 ± 0.03	12.1 ± 1.64
sh1-GCM-311	80	1.78 ± 0.12	0.44 ± 0.07	29.8 ± 2.9
GCM-411	-	0.99 ± 0.01	0.79 ± 0.01	146 ± 8
sh1-GCM-411	r.t.	0.82 ± 0.07	0.20 ± 0.03	26.2 ± 3.9
sh1-GCM-411	80	1.79 ± 0.06	0.45 ± 0.03	32.0 ± 2.6
sh2-GCM-411	80	1.83 ± 0.03	0.30 ± 0.01	19.1 ± 1.4
sh3-GCM-411	80	1.82 ± 0.05	0.26 ± 0.02	15.0 ± 1.1
GCM-511	-	1.05 ± 0.13	0.64 ± 0.06	120 ± 28
sh1-GCM-511	r.t.	0.98 ± 0.06	0.18 ± 0.02	19.2 ± 2.2
sh1-GCM-511	80	1.18 ± 0.05	0.32 ± 0.07	32.8 ± 11.4
GCD-411	-	2.37 ± 0.04	0.85 ± 0.04	66.9 ± 6.2
sh1-GCD-411	r.t.	1.56 ± 0.10	0.32 ± 0.06	25.2 ± 3.6
sh1-GCD-411	80	1.78 ± 0.06	0.53 ± 0.07	43.4 ± 10.6
sh2-GCD-411	80	1.69 ± 0.06	0.49 ± 0.04	39.3 ± 2.5
sh3-GCD-411	80	1.77 ± 0.16	0.43 ± 0.03	30.0 ± 0.9
